# Supplementary material for: ARID1A stabilizes non-homologous end joining factors at DNA breaks induced by the G4 ligand pyridostatin
Source: Cell Rep. 2025 Sep 11;44(9):116277. doi: 10.1016/j.celrep.2025.116277 (PMC12457572; doi:10.1016/j.celrep.2025.116277)
Supplement: Document S1. Figures S1–S6 [file mmc1.pdf]

Cell Reports, Volume *44*

Supplemental information

ARID1A stabilizes non-homologous end joining factors at DNA breaks induced by the G4 ligand pyridostatin

Susana Simões-Sousa, Noa Amin, Karen A. Lane, Alison Harrod, Malin Pedersen, Mercedes Pardo, Christine Rasetti-Escargueil, Zuzanna Kozik, Katheryn A.G. Begg, Alison Ribeiro, Susana Banerjee, Manuel Stucki, Kevin J. Harrington, Jyoti S. Choudhary, and Jessica A. Downs

Supplemental Figure 1

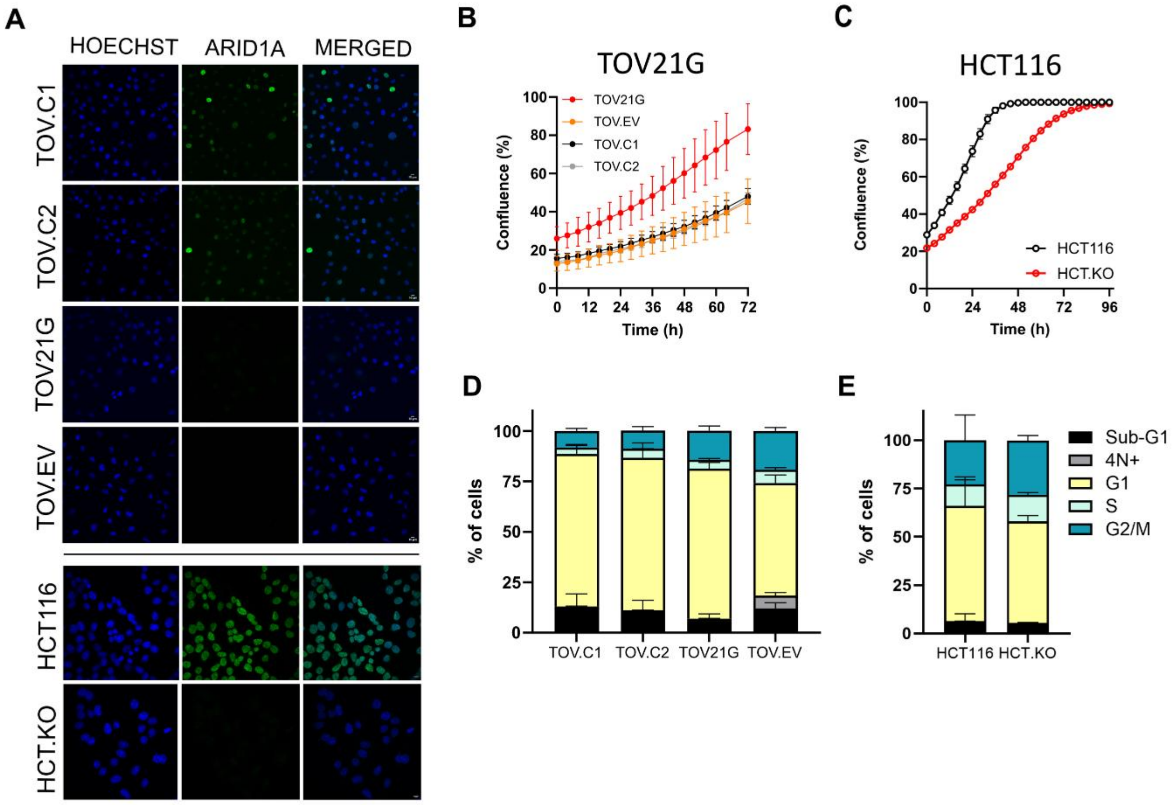

Supplemental Figure 1. ARID1A expression and its impact on cell proliferation and cell cycle profiles. Related to Figure 1. **(A)** Immunofluorescence staining of ARID1A (green) and Hoechst (blue) in TOV21G and HCT116 cell lines and derivatives. **(B)-(C)** Real-time confluence measurement of TOV21G (B) and HCT116 (C) isogenic cell lines, over 72 and 96 hours in untreated conditions, respectively. Data represents independent biological replicates, n = 3, mean ± SEM. **(D)-(E)** Cell cycle distribution assessed by flow cytometry using DNA content profiling of TOV21G (D) and HCT116 (E) isogenic cell lines in untreated conditions. Data represents independent biological replicates, n = 3, mean ± SEM.

Supplemental Figure 2

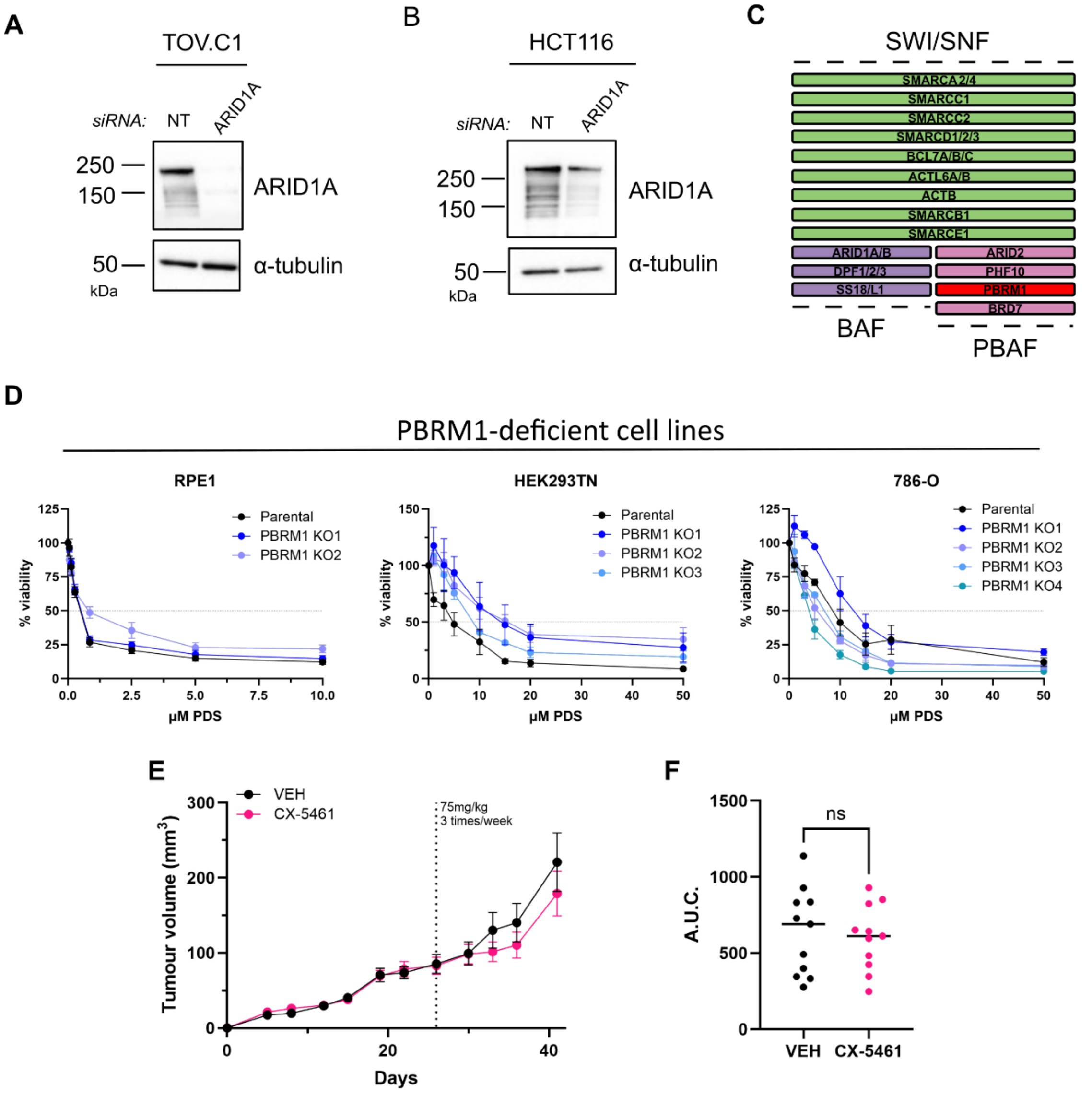

Supplemental Figure 2. Impact of PBRM1 modulation in response to G4 stabilisation and *in vivo* response of ARID1A-deficient tumours to treatment with clinical G4 ligand. Related to Figure 2. **(A)-(B)** Immunoblot showing ARID1A siRNA-mediated knockdown efficiency in ARID1A-proficient cell lines in TOV21G (A) and HCT116 (B). α-tubulin was used as loading control. **(C)** Schematic of the mammalian SWI/SNF chromatin remodelling complexes BAF and PBAF, highlighting PBRM1, an exclusive component of PBAF, in red. **(D)** Dose-response curves showing sensitivity to PDS in PBRM1-deficient cell lines derived from RPE1, HEK293 and 786-O cell lines. Data represents independent biological replicates, n = 5, 3, and 2, respectively, mean ± SEM. **(E)** Tumour growth curve of mice bearing TOV21G xenografts treated with vehicle (VEH) or CX-5461 under Treatment Regimen 2 (see schematic in Figure 2I). *n*=11 mice for each condition. **(F)** Area under the curve (A.U.C.) analysis of tumour volume over time from (E). Data were analysed by unpaired t-tests (ns, not significant).

Supplemental Figure 3

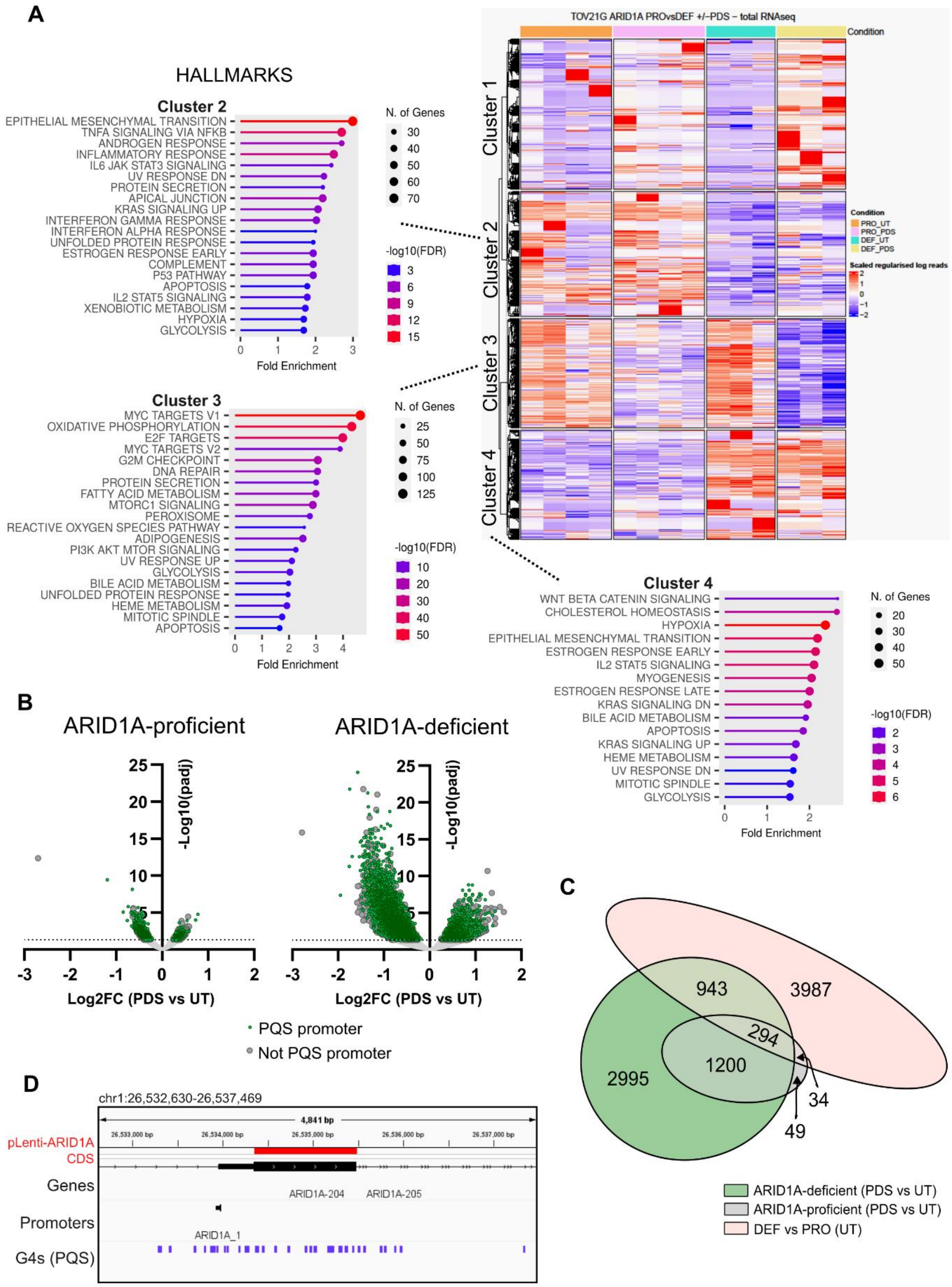

Supplemental Figure 3. ARID1A status differentially impacts transcriptional responses to G4 ligands. Related to Figure 3. **(A)** Heatmap showing the scaled regularised log-transformed read counts of the whole transcriptome of ARID1A-proficient and -deficient TOV21G isogenic cell lines, untreated or treated with PDS (10 μM, 4 hours), with samples split by condition and genes split into 4 clusters using *k*-means clustering. Hallmarks gene set enrichment analysis was carried out on these clusters, using ShinyGO v0.82. Cluster 1 did not have any significantly enriched gene sets. **(B)** Volcano plots showing differential gene expression induced by PDS treatment (10 μM, 4 hours), in ARID1A-proficient and -deficient cells (as in Fig 3A), highlighting significantly changed genes with promoter proximal to PQS (green) or promoters without PQS (grey). **(C)** Venn diagram comparing significantly changed genes upon PDS treatment (10 μM, 4 hours) in ARID1A-deficient (green) and -proficient (grey) TOV21G isogenic cells, compared with significantly changed genes in untreated ARID1A-deficient versus ARID1A-proficient TOV21G cell lines (pink). **(D)** Genome track of ARID1A gene region, showing promoter region (ARID1A_1) and predicted G4s (purple). Coding sequence (CDS) of the stably expressed pLenti-ARID1A construct starts in the region highlighted in red.

Supplemental Figure 4

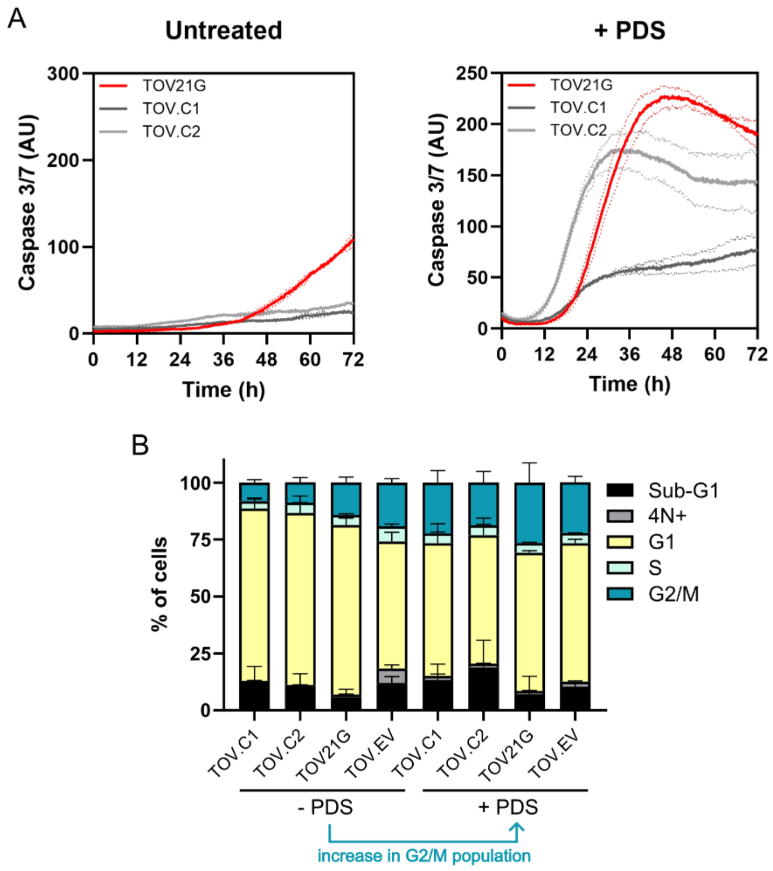

Supplemental Figure 4. ARID1A loss promotes caspase 3/7 activation, proliferation defects and cell cycle disruption following PDS exposure. Related to Figure 4. **(A)** Real-time measurement of caspase 3/7 activity in TOV21G, TOV.C1 and TOV.C2 cells over 72 hours in untreated conditions (left) or following PDS treatment (10 μM; right). Data represents independent biological replicates (n = 2, mean ± SEM, AU=arbitrary units). **(B)** Cell cycle distribution in TOV21G-derived cell lines under basal and PDS-treated conditions (10 μM, 96h hours), assessed by flow cytometry measuring DNA content. Data represents independent biological replicates (n = 3, mean±SEM).

Note that untreated samples are the same represented in Fig.S1C, which were run in parallel with the PDS treated samples represented in this figure.

Supplemental Figure 5

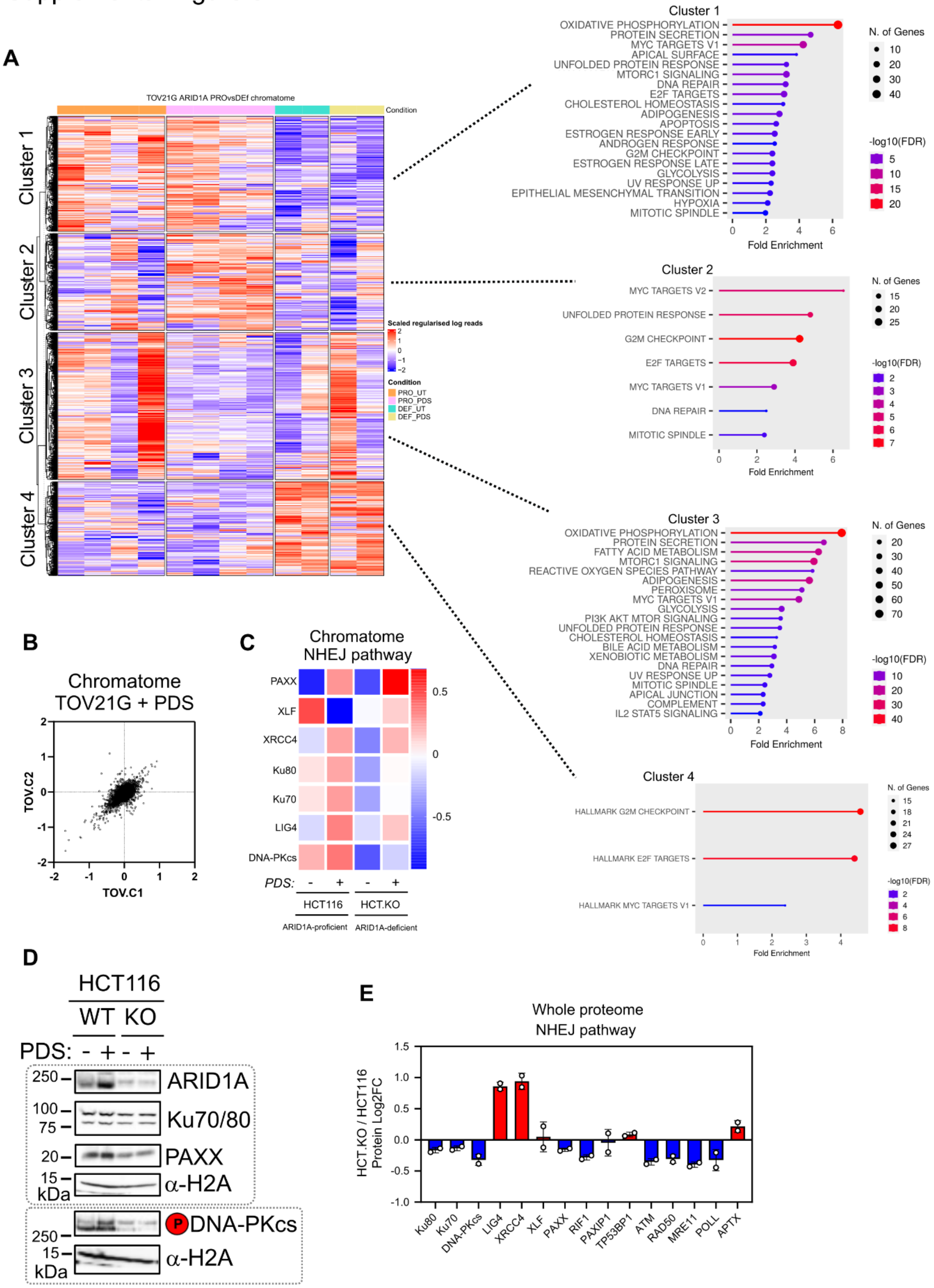

Supplemental Figure 5. ARID1A status impacts protein chromatin association patterns following PDS exposure. Related to Figure 5. **(A)** Heatmap of the log2-centred abundance of all chromatin-bound proteins across ARID1A-proficient and -deficient TOV21G cell lines, untreated or treated with PDS (10μM, 4 hours). Hierarchical clustering reveals distinct chromatin recruitment patterns in response to ARID1A status and PDS exposure. **(B)** Scatterplot comparing chromatin-associated proteome between TOV.C1 and TOV.C2 following PDS treatment (10 μM, 4 hours). **(C)** Heatmap of NHEJ components from chromatin-associated proteome in HCT.KO versus HCT116 parental cells. **(D)** Whole proteome quantification of NHEJ components in HCT.KO versus HCT116 parental cells, untreated or treated with PDS (20 μM, 4 hours). Points correspond to independent biological replicates, n = 2, mean ± SEM. **(E)** Immunoblot analysis of chromatin fractions from HCT116 parental and KO cells, untreated or treated with PDS (20 μM, 4 hours). α-H2A was used as a loading control.

Supplemental Figure 6

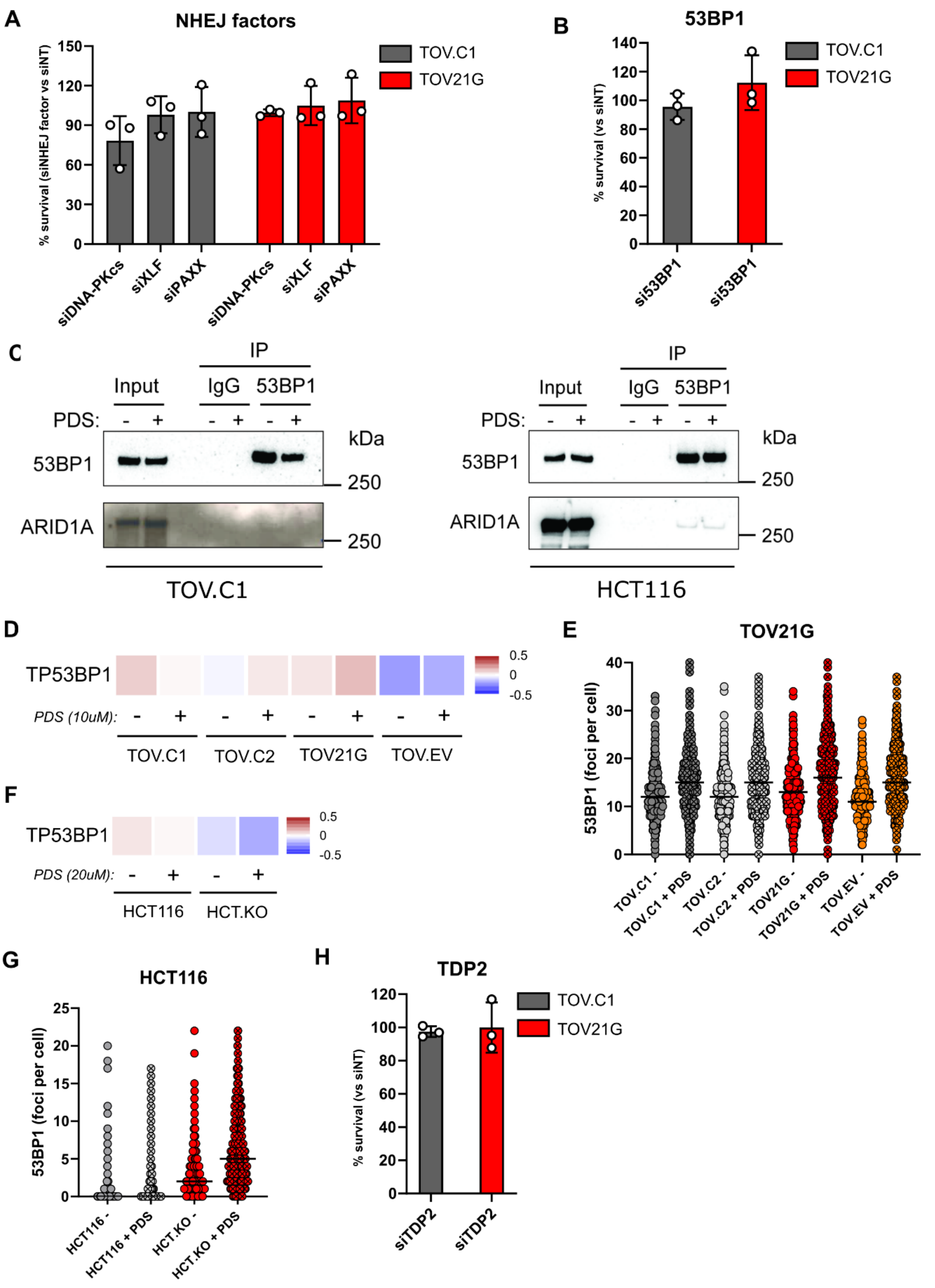

Supplemental Figure 6. Analysis of 53BP1 and NHEJ factors with ARID1A. Related to Figure 6. **(A)** Bar graph showing survival of TOV.C1 and TOV21G parental cells upon depletion of core NHEJ factors, compared to a non-targeting control (siNT) in the absence of treatment. Points correspond to independent biological replicates, n = 3, mean ± SEM. **(B)** Bar graph showing survival of TOV.C1 and TOV21G parental cells upon knockdown of 53BP1, compared to siNT, in the absence of treatment. Points correspond to independent biological replicates, n = 3, mean ± SEM. **(C)** Co-immunoprecipitation of 53BP1 and ARID1A. 53BP1 was immunoprecipitated from cell extracts prepared from untreated or PDS-treated (10 µM, 4 hours) TOV.C1 cells (left panel) or from untreated or PDS-treated (20 µM, 4 hours) HCT116 cells (right panel). IgG was used as a negative control. **(D)** Heatmap showing 53BP1 (TP53BP1) enrichment in chromatin in untreated or PDS-treated (10 µM, 4 hours) TOV21G isogenic cell lines. **(E)** Quantification of 53BP1 foci per cell by immunofluorescence in TOV21G derived cell lines, either untreated or treated with PDS (10 µM, 6 hours). Points represent number of foci in a single nucleus, line represents the median of all foci. **(F)** Heatmap showing 53BP1 (TP53BP1) enrichment in chromatin in untreated and PDS-treated (20 µM, 4 hours) HCT116 parental and ARID1A knockout cells. **(G)** Quantification of 53BP1 foci per cell by immunofluorescence in HCT116 parental and ARID1A knockout cell lines, either untreated or treated with PDS (20 µM, 6 hours). Points represent number of foci in a single nucleus, line represents the median. **(H)** Bar graph showing survival of TOV.C1 and TOV21G parental cells upon depletion of TDP2, compared to siNT, under untreated conditions. Points correspond to independent biological replicates, n = 3, mean ± SEM.

All siRNAs were used in parallel in each of the three biological replicates, hence the non-targeting siRNA controls are the same for each set of siRNAs represented in A, B and H.
